# Recurrent Multivalvular *Staphylococcus Lugdunensis* Endocarditis Causing Complete Heart Block after TAVR

**DOI:** 10.1155/2021/5334088

**Published:** 2021-11-01

**Authors:** Preeti Singhal, Somsupha Kanjanauthai, Wilson Kwan

**Affiliations:** ^1^Department of Internal Medicine, Keck School of Medicine of USC, Los Angeles, CA, USA; ^2^Los Angeles County + University of Southern California Medical Center, Los Angeles, CA, USA; ^3^Department of Cardiovascular Medicine, Keck School of Medicine of USC, Los Angeles, CA, USA

## Abstract

Prosthetic valve endocarditis after transcatheter aortic valve replacement (PVE after TAVR) is a feared complication most often observed during the early postprocedural period. We report a case of severe, multivalvular PVE after TAVR with complete heart block caused by an uncommon organism. A 78-year-old female with prior *Streptococcus agalactiae* mitral valve endocarditis treated with antibiotics presented one year later with severe, symptomatic aortic insufficiency. She subsequently underwent TAVR given high surgical risk. Six weeks post-TAVR, she presented with syncope, fever, and complete heart block. Transthoracic echocardiogram was not demonstrative of vegetation. Blood cultures were positive for *Staphylococcus lugdunensi*s. Transesophageal echocardiogram (TEE) demonstrated vegetations of the aortic, mitral, and tricuspid valves and aorto-mitral continuity. While awaiting surgery, the patient developed cardiac arrest; she was resuscitated and taken to surgery emergently. The patient underwent TAVR explantation, bovine pericardial tissue aortic and porcine bioprosthetic mitral valve replacements, and tricuspid valve repair. Additionally, left main coronary artery endarterectomy was performed due to presence of infectious vegetative material. *Staphylococcus lugdunensis* is an unusual but virulent organism that may damage both native and prosthetic valves. Early surgery is recommended for PVE after TAVR, especially in cases with perivalvular disease causing conduction abnormalities. *Learning Objectives.* TAVR has revolutionized the management of severe aortic stenosis and has even been successfully utilized in select cases of aortic regurgitation. Unfortunately, there are a number of associated complications that can be difficult to diagnose, such as prosthetic valve endocarditis (PVE). We emphasize maintaining a high clinical suspicion for PVE after TAVR in patients presenting with conduction abnormalities and highlight the importance of early surgical management in cases complicated by heart block, abscesses, or destructive penetrating lesions.

## 1. Introduction

Transcatheter aortic valve replacement (TAVR) has become the treatment of choice for those with severe aortic stenosis and can be an effective option for patients with severe aortic regurgitation and high surgical risk. However, with the increasing rate of TAVRs being performed comes a need to better understand TAVR-associated complications such as prosthetic valve endocarditis (PVE), leaflet thrombosis, and paravalvular regurgitation. Here, we emphasize having a high clinical suspicion for PVE after TAVR in patients presenting with nonspecific symptoms and conduction abnormalities, an uncommon sequela. We highlight the importance of early surgical intervention in patients with PVE after TAVR whose clinical courses are complicated by heart block, paravalvular abscesses, and destructive penetrating lesions.

## 2. Case Report

**Figure 1:**
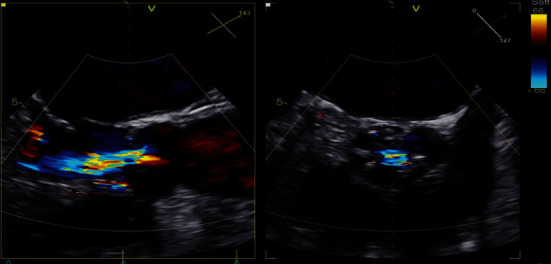
Transesophageal echocardiography image acquired prior to TAVR demonstrating thickened aortic valve with significant central aortic regurgitation without evidence of vegetation or perforation.

A 78-year-old woman with past medical history significant for hypertension, type 2 diabetes mellitus, May-Thurner syndrome managed with iliac vein stenting, lower extremity lymphedema status post lymphovenous bypass, *Streptococcus agalactia*e endocarditis of the mitral valve treated with a six-week course of antibiotics with posttreatment TEE showing resolution of infection, and severe, symptomatic, degenerative aortic regurgitation (Figure 1) status post uncomplicated TAVR (Edwards Lifesciences, Irvine, CA) given high surgical risk with postprocedure TTE showing peak and mean gradients of 27 mmHg and 12 mmHg, respectively, across the valve and no perivalvular regurgitation, presented six weeks post-TAVR after a syncopal episode. The patient reported several days of nausea, fevers, dizziness, and malaise.

On examination, her temperature was 38.7°C, heart rate 40/min, respiratory rate 19/minute, blood pressure 100/52 mmHg, and oxygen saturation 95% on room air. The patient was oriented but uncomfortable and lethargic appearing. Cardiovascular examination revealed an audible S1/S2 with a grade III/VI systolic ejection murmur. She also had bilateral lower extremity edema and crackles in bilateral lung bases. Electrocardiogram demonstrated new complete heart block. Transvenous pacing was initiated in the emergency department.

Laboratory studies revealed a leukocyte count of 7.0 K/cumm (reference range: 4.5-10), platelet count 18 K/cumm (reference range: 160-360), blood glucose 446 mg/dL, alkaline phosphatase 254 U/L (reference range: 35-104), and pro-BNP 3886 pg/mL (reference range: <450). Troponin-T, lactate, and TSH were within normal limits. A rapid SARS-CoV-2 assay was negative. Chest radiography demonstrated an enlarged cardiomediastinal silhouette with pulmonary vascular congestion. TTE showed a LVEF of 55%, normal right ventricular systolic function, and a well-seated bioprosthetic aortic valve with trace intravalvular aortic regurgitation without paravalvular leak (peak velocity 3.3 m/s, mean gradient 26 mmHg, and effective orifice area 1.2 cm^2^). No vegetations or abscesses were visualized. Blood cultures were positive for *Staphylococcus lugdunensis*.

**Figure 2:**
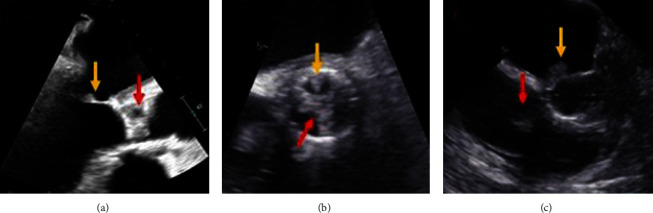
Transesophageal echocardiography of infected TAVR. (a) Thickened TAVR leaflets (red arrow) and vegetation of the mitral valve (yellow arrow). (b) Thickened TAVR leaflets (red arrow) and vegetation of the aortic cusp (yellow arrow). (c) Vegetations of the tricuspid valve (red arrow) and aortomitral continuity (yellow arrow).

**Figure 3:**
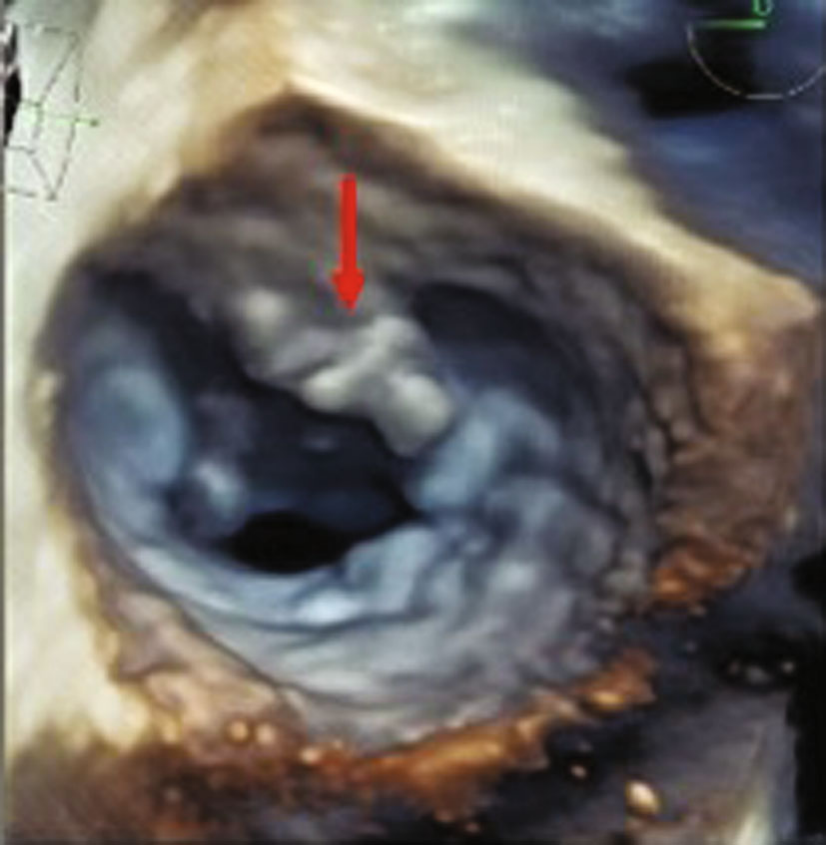
3D transesophageal echocardiography of infected TAVR showing bulky vegetation on the anterior mitral valve leaflet (arrow).

The patient was initiated on broad-spectrum antibiotics with vancomycin and piperacillin/tazobactam. Blood cultures remained positive for *Staphylococcus lugdunensis* for three consecutive days. Given persistent bacteremia and strong clinical suspicion for PVE following TAVR, antibiotic therapy was transitioned to vancomycin, gentamycin, and rifampin. TEE was performed on hospital day 3 and demonstrated bulky vegetative material extending from the aortic root to the atrial surface of the anterior mitral leaflet measuring 2.0 × 0.07 cm, as well as small discrete vegetative lesions measuring 0.5 × 0.3 cm and 0.5 × 0.3 cm on the atrial side of the anterior mitral leaflet and ventricular side of the aortic valve, respectively (Figure 2(a)–2(c) and Figure 3), moderate mitral regurgitation, trivial bioprosthetic aortic valve regurgitation, mild bioprosthetic aortic valve stenosis (peak velocity: 3.3 m/s) with moderately thickened leaflets along all three coaptation lines suggestive of vegetations, and mild tricuspid regurgitation. Antibiotics were narrowed to gentamycin, oxacillin, and rifampin based on blood culture susceptibilities. Surgical evaluation was initiated, and the patient was transferred to an outside hospital for further medical management and operative planning.

**Figure 4:**
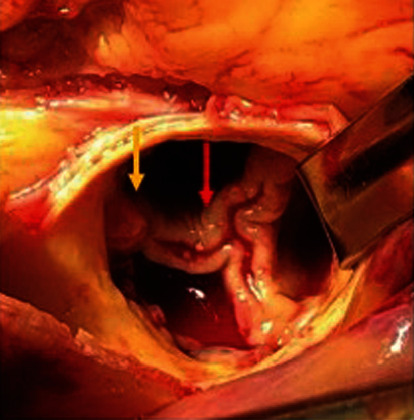
Intraoperative view of TAVR showing thickened TAVR leaflets (red arrow) and vegetative material (yellow arrow).

On hospital day 10, the patient developed ventricular tachycardia with cardiac arrest and was successfully resuscitated after one round of cardiopulmonary resuscitation. She was taken for emergent surgery, undergoing TAVR valve explantation (Figure 4), bovine pericardial tissue aortic and porcine bioprosthetic mitral valve replacement, tricuspid valve repair with anterior septal commissuroplasty, ventricular septal defect closure with a bovine pericardial patch, and right ventricular free wall perforation repair, likely a complication from transvenous pacemaker placement versus secondary to recent infarct from septic emboli. Additionally, ostial left main coronary artery endarterectomy was performed given intraoperative findings of near-total occlusion of the artery with clot and vegetative material, the likely etiology for the patient's cardiac arrest. A postintervention intraoperative transesophageal echocardiogram showed a well-seated and properly functioning bioprosthetic aortic valve with no obvious intra- or perivalvular regurgitation, a well-seated and properly functioning bioprosthetic mitral valve with trace intravalvular regurgitation and no definitive evidence of a perivalvular leak, mild tricuspid regurgitation, no evidence of residual VSD, and an ejection fraction preserved to prebypass baseline. Final surgical pathology report of the tissue from the aortic valve prosthesis, mitral valve, and left main endarterectomy showed organizing fibrin and prominent acute inflammation with gram stain negative for bacteria.

The patient's postoperative course was complicated by acute on chronic biventricular failure requiring inotropic and mechanical circulatory support, vasodilatory shock, and renal failure requiring continuous renal replacement therapy. After extensive discussions with the patient's family, a decision was made to change her code status to do not resuscitate. She passed seven days postoperatively from multiorgan failure.

## 3. Discussion

TAVR has become the treatment of choice for patients with severe aortic stenosis and can be an option for those with severe aortic regurgitation who are deemed high surgical risk. TAVR is associated with a unique set of complications [1, 2]. Albeit uncommon (incidence: 1-3%), PVE following TAVR is associated with an in-hospital mortality rate of 36% and a 2-year mortality of 67% according to a large 250 case registry [1, 3]. The risk of PVE is highest in the first year after TAVR; the most common organisms implicated overall are *Streptococcus spp.* (28.9%), followed by *Enterococcus spp.* (26.2%), and *Staphylococcus aureus* (21.5%) [2]. *Staphylococcus lugdunensis* is a relatively uncommon but highly virulent coagulase negative staphylococcus that is notable for its production of and adherence to biofilm, enabling growth on bioprosthetic materials and native tissues [4, 5].

Conduction disturbances occur in 4-10% of patients with IE. Those with aortic valve involvement are at higher risk of developing heart block due to the proximity of the His-Purkinje system to the aortic valve [6]. Recurrent IE is relatively uncommon; in a multicenter study of 1874 patients with IE, 91 (4.8%) had a documented episode of repeat IE, of which 74 (81%) involved a new bacterial species [7]. 16 cases (18%) involved a prosthetic valve; the causative organism was different between initial and recurrent infections in all 16 cases [7]. Repeat IE was associated with intravenous drug use and hemodialysis dependence, neither of which were characteristic of the patient in the case [7].

Although TTE and TEE are the cornerstones for diagnosis of endocarditis, echocardiographic detection of PVE after TAVR can be challenging in comparison to native valve endocarditis or PVE after SAVR [1]. Contributing factors include the absence of a surgical sewing ring or decalcification area, remnant native calcified aortic valve, and valve struts, all of which may cause significant echocardiographic artifacts and obscure visualization of the prosthetic valve [1]. Additionally, free space between the transcatheter and native aortic valve also presents a challenge for the diagnosis of vegetation or abscess by TEE [1].

Conservative management of PVE after TAVR is often inadequate and requires surgical valve replacement. [8] The American Heart Association and American College of Cardiology recommends early surgery during initial hospitalization prior to the completion of antibiotics in patients with PVE complicated by heart block, annular or aortic abscess, or destructive/penetrating lesions [1, 9]. In cases of uncomplicated PVE with a sensitive organism, early surgery is not required but should be considered if complications arise [1]. Our patient's significant conduction disease, multivalvular and aortic root involvement, and acute decompensation necessitated aggressive surgical management.

This case highlights the importance of prompt recognition of clinical signs of PVE, particularly when echocardiographic diagnosis is challenging. The presentation with complete heart block and intraoperative findings of widespread tissue involvement and left main coronary obstruction by vegetative material highlight the pathogenicity of *Staphylococcus lugdunensis—*an uncommon but virulent organism. Although we suspected PVE given the patient's history of prior *Streptococcus agalactiae* endocarditis and TAVR, we were surprised to find a different causative organism. It remains unclear how the patient acquired *S. lugdunensis* bacteremia. Early surgical intervention is recommended for PVE after TAVR when complicated by heart block, abscesses, or penetrating lesions given the propensity for acute decompensation.

## Data Availability

This is a case report; therefore, all data were obtained from the patient's clinical course and references listed in the manuscript.

## Abbreviations

TAVR: Transcatheter aortic valve replacement
TEE: Transesophageal echocardiogram
TTE: Transthoracic echocardiogram
PVE: Prosthetic valve endocarditis
LVEF: Left ventricular ejection fraction.
